# Supplementary material for: Causal Relationship between Aging and Anorexia Nervosa: A White-Matter-Microstructure-Mediated Mendelian Randomization Analysis
Source: Biomedicines. 2024 Aug 16;12(8):1874. doi: 10.3390/biomedicines12081874 (PMC11351342; doi:10.3390/biomedicines12081874)
Supplement: Supplementary file 1 [file biomedicines-12-01874-s001.zip › Supplementary Figure/Figure S4/Singe SNP plot of MR analysis for TL to ubm-b-1577.pdf]

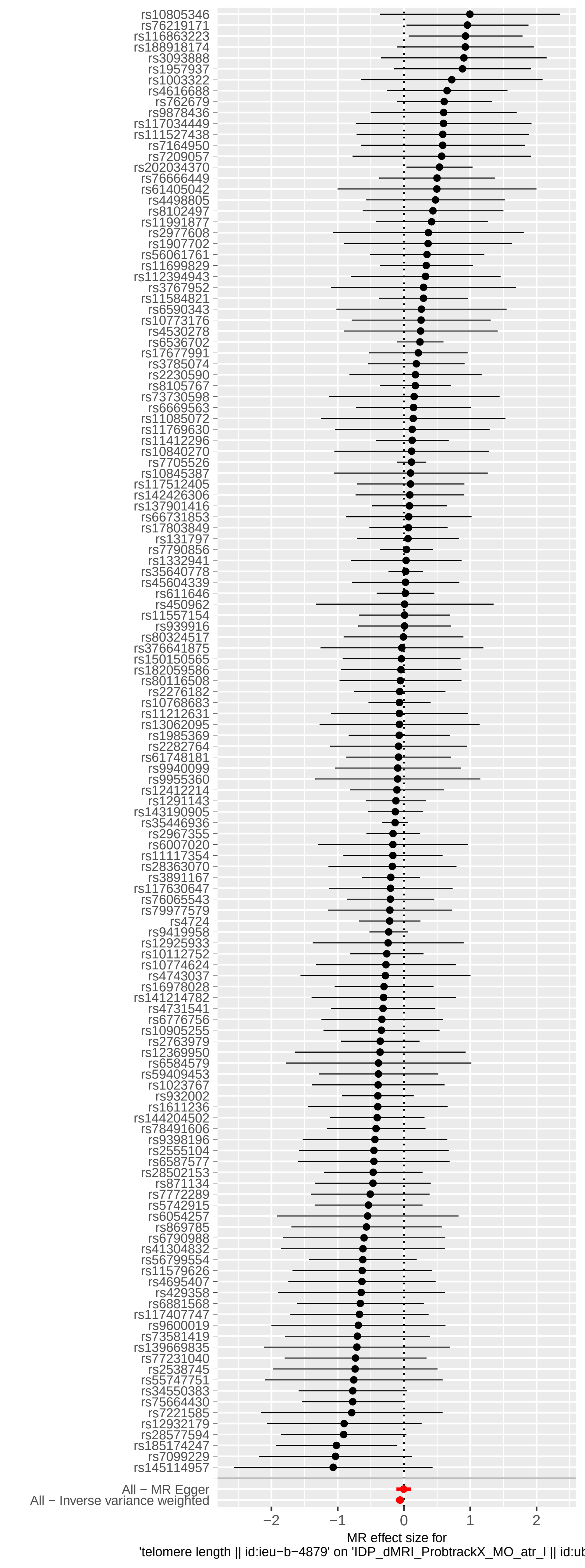
rs10805346
rs76219171
rs116863223
rs188918174
rs3093888
rs1957937
rs1003322
rs4616688
rs762679
rs9878436
rs117034449
rs111527438
rs7164950
rs7209057
rs202034370
rs76666449
rs61405042
rs4498805
rs8102497
rs11991877
rs2977608
rs1907702
rs56061761
rs11699829
rs112394943
rs3767952
rs11584821
rs6590343
rs10773176
rs4530278
rs6536702
rs17677991
rs3785074
rs2230590
rs8105767
rs73730598
rs6669563
rs11085072
rs11769630
rs11412296
rs10840270
rs7705526
rs10845387
rs117512405
rs142426306
rs137901416
rs66731853
rs17803849
rs131797
rs7790856
rs1332941
rs35640778
rs45604339
rs611646
rs450962
rs11557154
rs939916
rs80324517
rs376641875
rs150150565
rs182059586
rs80116508
rs2276182
rs10768683
rs11212631
rs13062095
rs1985369
rs2282764
rs61748181
rs9940099
rs9955360
rs12412214
rs1291143
rs143190905
rs35446936
rs2967355
rs6007020
rs11117354
rs28363070
rs3891167
rs117630647
rs76065543
rs79977579
rs4724
rs9419958
rs12925933
rs10112752
rs10774624
rs4743037
rs16978028
rs141214782
rs4731541
rs6776756
rs10905255
rs2763979
rs12369950
rs6584579
rs59409453
rs1023767
rs932002
rs1611236
rs144204502
rs78491606
rs9398196
rs2555104
rs6587577
rs28502153
rs871134
rs7772289
rs5742915
rs6054257
rs869785
rs6790988
rs41304832
rs56799554
rs11579626
rs4695407
rs429358
rs6881568
rs117407747
rs9600019
rs73581419
rs139669835
rs77231040
rs2538745
rs55747751
rs34550383
rs75664430
rs7221585
rs12932179
rs28577594
rs185174247
rs7099229
rs145114957
All − MR Egger
All − Inverse variance weighted
−2
−1
0
1
2
MR effect size for
'telomere length || id:ieu−b−4879' on 'IDP_dMRI_ProbtrackX_MO_atr_l || id:ub
